# Right inferior frontal gyrus theta-burst stimulation reduces smoking behaviors and strengthens fronto-striatal-limbic resting-state functional connectivity: a randomized crossover trial

**DOI:** 10.3389/fpsyt.2023.1166912

**Published:** 2023-06-28

**Authors:** Spencer Upton, Alexander A. Brown, Mojgan Golzy, Eric L. Garland, Brett Froeliger

**Affiliations:** ^1^Department of Psychological Sciences, University of Missouri, Columbia, MO, United States; ^2^Department of Psychiatry, School of Medicine, University of Missouri, Columbia, MO, United States; ^3^Cognitive Neuroscience Systems Core Facility, University of Missouri, Columbia, MO, United States; ^4^Biostatistics Unit, Department of Family and Community Medicine, School of Medicine, University of Missouri, Columbia, MO, United States; ^5^Center on Mindfulness and Integrative Health Intervention Development, College of Social Work, University of Utah, Salt Lake City, UT, United States

**Keywords:** addiction, smoking, tobacco, craving, repetitive transcranial magnetic stimulation, resting state fMRI, right inferior frontal gyrus (rIFG)

## Abstract

**Introduction:**

Functional and anatomical irregularities in the right inferior frontal gyrus (rIFG), a ventrolateral prefrontal region that mediates top-down inhibitory control over prepotent behavioral responding, are implicated in the ongoing maintenance of nicotine dependence (ND). However, there is little research on the effects of neuromodulation of the rIFG on smoking behavior, inhibitory control, and resting-state functional connectivity (rsFC) among individuals with ND.

**Methods:**

In this double-blind, crossover, theta-burst stimulation (TBS) study, adults with ND (*N* = 31; female: *n* = 15) completed a baseline session and were then randomized to two counterbalanced sessions of functionally neuronavigated TBS to the rIFG: continuous TBS (cTBS) on 1 day and intermittent TBS (iTBS) on another. Differences in cigarette cravings, smoking, and fronto-striatal-limbic rsFC were assessed.

**Results:**

Relative to baseline, cTBS significantly reduced appetitive and withdrawal cravings immediately after treatment. The effects of cTBS on withdrawal craving persisted for 24 h, as well as produced a reduction in smoking. Furthermore, cTBS significantly strengthened rsFC between the rIFG pars opercularis and subcallosal cingulate (fronto-striatal circuit), and between the rIFG pars opercularis and the right posterior parahippocampal gyrus (fronto-limbic circuit). At post-24 h, cTBS-induced increase in fronto-striatal rsFC was significantly associated with less appetitive craving, while the increase in fronto-limbic rsFC was significantly associated with less withdrawal craving and smoking.

**Discussion:**

These findings warrant further investigation into the potential value of rIFG cTBS to attenuate smoking behavior among individuals with ND.

## Introduction

Chronic use of addictive drugs, such as nicotine, results in maladaptive goal-directed behaviors and modifies neural circuitry subserving motivation and executive function (1–3). Relative disturbances in response inhibition and salience attribution to drug cues represent two core factors maintaining smoking behaviors among individuals with nicotine dependence (4–6). The majority of adults that smoke relapse when attempting to quit, even when using first-line FDA-approved cessation products (7). Furthermore, adults that smoke cite disturbances in cognition (8, 9) and craving (8, 10, 11) as primary factors that precipitate relapse. Thus, there is an urgent need to develop innovative strategies for treating deficits in core neurocognitive domains to improve smoking cessation outcomes (12).

Functional magnetic resonance imaging (fMRI) research with individuals with substance use disorders (SUDs) has informed neural circuit-based models for treating addiction pathophysiology (13–18). The extant literature supports a model whereby impaired response inhibition and salience attribution to drug cues are mediated by a common top-down modulatory influence from the ventrolateral prefrontal cortex (vlPFC) [i.e., right inferior frontal gyrus (rIFG)] (19). This model is supported by SUD studies associating rIFG dysfunction with deficits in both inhibitory control (IC) (9, 17, 19–32) and the proactive regulation of craving (19, 25, 33–36). IC refers to the ability to disrupt and withhold a prepotent response (37), while the regulation of craving refers to the ability to modify motivational responses to conditioned drug cues (33). Furthermore, systems neuroscience research assessing resting-state functional connectivity (rsFC) (38) has demonstrated that dysregulated connectivity between the rIFG, striatum, and limbic reward structures (henceforth, fronto-striatal-limbic circuitry) may underlie the capacity to exert top-down cognitive control over motivated behavioral responding (39).

Among individuals with a SUD, dysregulated fronto-striatal-limbic rsFC has been widely reported in the literature and associated with impulsivity (39) and craving (38, 40, 41). Weakened fronto-striatal-limbic rsFC has been reported among individuals with dependence on nicotine (42–45), cocaine (46, 47), and opioids (48), as well as those with addictive behaviors such as internet gaming disorder (49) and problematic smartphone use (50). Though it is not clear whether weaker fronto-striatal-limbic rsFC is a consequence of addiction or a predisposing risk factor for developing a substance or behavioral addiction, the extant literature suggests rsFC in fronto-striatal-limbic circuitry may serve as a treatment target to remediate dysregulated cognitive control over addictive behaviors. However, there remains a dearth of mechanistic research demonstrating the potential clinical value of using neuromodulation to target fronto-striatal-limbic circuitry to improve cessation outcomes.

Theta-burst stimulation (TBS), a patterned form of repetitive transcranial magnetic stimulation (rTMS), shows promise for treating addiction pathophysiology (51). The two common types of TBS are continuous TBS (cTBS) and intermittent TBS (iTBS). Early research that administered TBS to the motor cortex provided evidence to support a model where cTBS produced an inhibitory—long-term depression (LTD)-like effect; whereas iTBS produced an excitatory—long-term potentiation (LTP)-like effect (52, 53). Given the prior literature on the therapeutic value of administering excitatory-like rTMS patterns to the dorsolateral prefrontal cortex (dlPFC), at the start of this study in 2019, we initially hypothesized that excitatory-like rTMS (i.e., iTBS) to the vlPFC would produce a clinically relevant improvement in behavioral inhibition and smoking behavior, as compared to an inhibitory-like pattern (i.e., cTBS). However, during the time we conducted the study, evidence was published suggesting that the effects of TBS on the lateral prefrontal cortices may not correspond to a dissociable inhibitory or excitatory outcome, as once proposed (54). Moreover, cTBS to the right dlPFC has been recently shown to reduce anxiety symptoms (55) and those findings have been subsequently supported by a sham-controlled cTBS clinical trial for generalized anxiety disorder demonstrating that cTBS reduces anxiety (56). Furthermore, recent evidence suggests that the dissociable effects of TBS may also depend on pulse number (57). In sum, recent evidence has cast doubt on our original rationale, suggesting that either iTBS or cTBS to the vlPFC might be effective treatments for smoking cessation and we have analyzed and presented our findings in light of that evidence. Thus, there continues to remain a need for a principled evaluation of the neural and behavioral effects of both iTBS and cTBS on prefrontal-mediated cognitive control, and understanding mechanisms of action of TBS on drug use relevant behaviors.

The most common cortical target for examining the effects of neurostimulation on drug use behaviors is the left dlPFC—an anatomical target adopted from FDA-approved protocols for treating major depressive disorder and shown to improve smoking cessation outcomes (58). However, research examining the effects of neuromodulation over alternative cortical targets in individuals with SUDs remains scarce (59, 60). Given the role of the vlPFC (i.e., rIFG) in mediating IC and craving regulation, its strength of functional connectivity with striatal and limbic reward circuitry mediating drug-seeking behaviors, and its anatomical location being amenable to TBS, the rIFG is an ideal alternative cortical target for examining the potential therapeutic value of neuromodulation for treating addiction pathophysiology. Further support for stimulating the rIFG comes from a recent multisite double-blind sham-controlled randomized clinical trial that administered bilateral deep rTMS over the lateral prefrontal cortices in adults with nicotine dependence and found it reduced both smoking and craving (61). Despite this knowledge, there is a gap in the extant literature on the effects of TBS on the rIFG for modifying addictive behaviors. To address this gap in the literature and extend our previous research (17, 62), relative to a baseline session with no TBS, the current study examined the acute effects of functionally neuronavigated iTBS and cTBS to the rIFG at 80% resting motor threshold (RMT) on smoking behaviors and fronto-striatal-limbic rsFC.

## Materials and methods

### Participants

Participants (*N* = 31) (Table 1; Supplementary Table 1) were recruited from the local community via media outlets in Columbia-Missouri by research staff. Inclusion criteria were being an individual aged between 18 and 65 years; a minimum history of smoking ≥ 10 cigarettes per day (CPD) for ≥2 years; carbon monoxide level of ≥10 (Vitalograph Inc.); stable mental and physical health; and willingness to provide informed consent. Exclusion criteria were contraindication to MRI or TBS; use of substances that lower seizure threshold; history of disorders affecting the brain; unstable cardiac disease, uncontrolled hypertension, severe renal or liver insufficiency, or sleep apnea; current or past psychosis; breath alcohol > 0; or positive pregnancy test. The study was approved by the Institutional Review Board at the University of Missouri, Columbia.

**Table 1:** Demographics and clinical characteristics.

**Measure**	**Participants (*N* = 31)**
**Demographics**	
Participants (female)	31 (15)
Sex, female, *n* (%)	15 (48.4%)
Age, years, mean (SD)	47.7 (8.7)
**Race**, ***n*** **(%)**	
Black or African American	5 (16.1%)
Caucasian non-Latinx/Hispanic	25 (80.6%)
Multiple	1 (3.2%)
**Education**, ***n*** **(%)**	
No high school diploma	2 (6.5%)
High school diploma	6 (19.4%)
Some college	15 (48.4%)
4-year college degree	6 (19.4%)
Advanced degree	2 (6.5%)
**Household income, annually**, ***n*** **(%)**	
$16,000 or less	7 (22.6%)
$16,001–31,000	7 (22.6%)
$31,001–48,000	7 (22.6%)
$48,001–64,000	2 (6.5%)
$64,001–80,000	1 (3.2%)
$80,001–96,000	4 (12.9%)
$96,001 or more	2 (6.5%)
Not reported	1 (3.2%)
**Clinical characteristics, mean (SD)**	
Nicotine dependence, FTND^a^	5.4 (2.1)
Daily cigarettes, past 30-days	18.4 (4.5)
Years smoking	29.8 (9.0)
Pack years	27.7 (10.7)
Impulsivity, BIS^b^ total	61.4 (9.5)
NoGo adjusted % correct, IC GGNG task^c^	44.8 (21.2)

^a^Fagerstrom Test for Nicotine Dependence (score range: 0–10).

^b^Barratt Impulsiveness Scale (score range: 30–120).

^c^Inhibitory control *GoGo/NoGo* task (score range: 0–100).

### Design overview

The study's aims and analyses were part of a larger TBS trial in individuals with nicotine dependence (ClinicalTrials.gov Identifier: NCT03960138) (Supplementary Table 1). Based on the extant literature supporting the clinical value of high-frequency rTMS to left dlPFC for depression (58, 63), we initially hypothesized that iTBS would result in clinically relevant improvements as compared to baseline and cTBS. However, during the course of the study, evidence was published (54–57) that casted doubt on this rationale and suggested that either iTBS or cTBS might result in clinically relevant improvements. Thus, the current study examining the effects of i/cTBS on brain and smoking behavior was exploratory.

**Figure 1:**
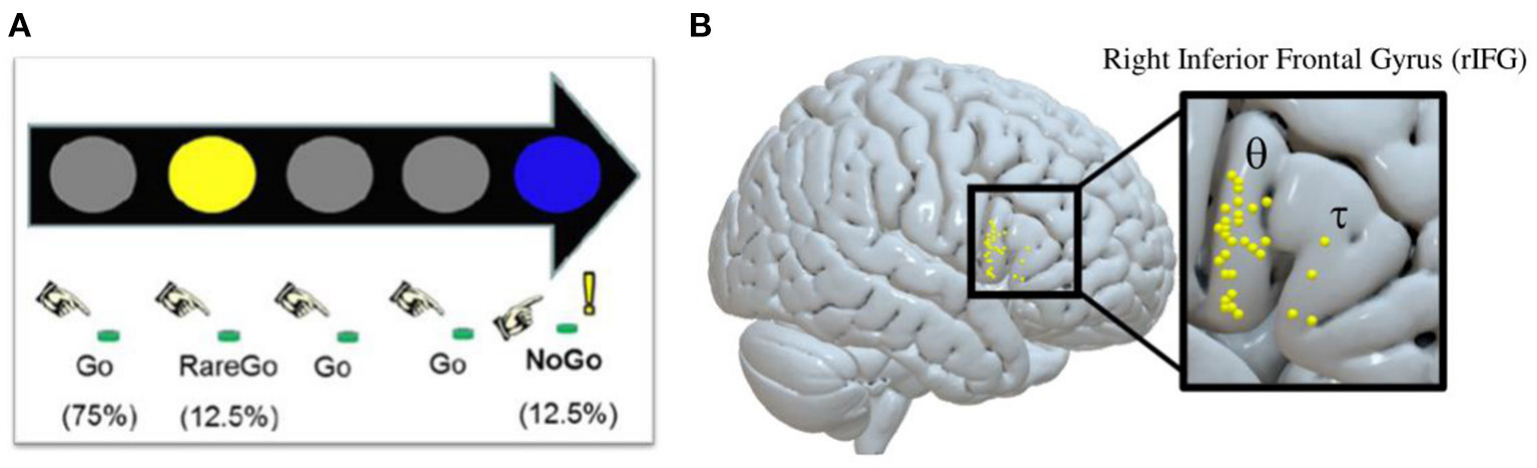
Inhibitory control GoGo/NoGo (GGNG) task and corresponding functionally neuronavigated theta-burst stimulation (TBS) targets identified at baseline for each participant. **(A)** Depiction of the inhibitory control GGNG task. **(B)** Depiction of the peak GGNG task-related blood-oxygenation level-dependent (BOLD) response in the right interior frontal gyrus (rIFG) during successful inhibitory control trials, while controlling for lapses in attention and novelty detection for each participant [θ = rIFG pars opercularis (*n* = 27); τ = rIFG pars triangularis (*n* = 4)]. The majority of peak BOLD responses lie on the rIFG pars opercularis (rIFGoper).

Following informed consent, participants attended a screening and training session, which included an MRI mock scan and acclimation to the TBS equipment. Eligible participants went on to complete three additional sessions each separated by at least 48 h. Session one was a baseline session, which utilized an IC GoGo/NoGo (GGNG) task (described below) during fMRI to determine each participant's rIFG target for neuronavigation-guided TBS at the following sessions. Successful IC, controlling for novelty detection, elicits activation within the rIFG, particularly the pars opercularis subregion (27, 64). For each participant, their peak rIFG IC BOLD cluster was set as the functional target for the following TBS sessions (Figure 1). Next, participants attended two randomized, counterbalanced, neuronavigated TBS sessions to the rIFG—one administering cTBS, and the other administering iTBS.

Participants were randomized to treatment orders with an allocation ratio of 2:2 in blocks of 4, which was concealed by non-research staff. Participants and research staff collecting data were blinded to treatment orders. All TBS treatments were administered by a dedicated TBS technician who was not involved with data collection. Self-reported electronic questionnaires on cravings and side effect symptoms were collected at the start and end of each session in the laboratory, while electronic questionnaires on cravings, smoking, and side effects were collected remotely 24 h following each session by smartphone. Resting-state fMRI was collected at baseline and 20 min after each TBS treatment. At the end of the last session, participants and researchers that collected data completed a study blind assessment. To control nicotine satiety at the start of each session, participants were encouraged to smoke immediately before coming into the laboratory. No significant differences in session-start carbon monoxide (CO) levels or cravings were detected, which provided confirmation that participants started each session with equivalent levels of nicotine satiety (Supplementary Table 2). By the end of each laboratory session, participants had not smoked for ~2 h.

### Magnetic resonance imaging

#### Image acquisition

Whole-brain images were acquired using a 3T Siemens Prisma Fit MRI scanner. A T1-weighted magnetization prepared—rapid gradient echo (MPRAGE) sequence (TR = 2,300 ms, TE = 2.26 ms, FA = 8°, 192 ascending slices, 1 mm^3^ voxels, FOV = 256 mm, phase encoding direction = A >> P) was used to acquire anatomical images. Functional T2^*^-weighted images were acquired to measure BOLD responses using a simultaneous multi-slice echo-planar imaging (EPI) sequence (acceleration factor = 3, TR = 2,000 ms, TE = 36 ms, FA = 70°, 69 interleaved slices, 2.2 mm^3^ voxels, FOV = 207 mm, phase encoding direction = A >> P).

#### Baseline fMRI GGNG IC task

At baseline, each participant performed an IC GGNG task (17, 65) during an fMRI scan (duration = 7.2 min; volumes = 216) to identify the rIFG treatment target for each participant. During the task, colored circles were presented in rapid succession with instructions to press a button with the right index finger in response to frequent gray circles (Go, 75.4%; *n* = 388) and rare yellow circles (RareGo, 12.5%; *n* = 65) and to withhold a response to rare blue circles (NoGo, 12.5%; *n* = 65). Random, infrequent presentation of NoGo trials facilitated prepotency of response. The inclusion of RareGo trials allowed for the determination of BOLD activation specific to IC after controlling for activation associated with novelty detection. Additionally, to control the effects of attentional lapses during the task, reported NoGo accuracy was adjusted to include only NoGo trials with a correct response to the preceding Go trial. All stimuli were presented for 400 ms and were followed by a 400 ms interval.

To increase the precision of TBS target identification, IC task images were processed in native space. Preprocessing consisted of structural cortical surface reconstruction (FreeSurfer); slice-timing correction and rigid-body head motion correction (FMRIB Software Library); coregistration (FreeSurfer); and the estimation and removal of noise components using an iterative sparse noise-modeling technique (66). Data were entered into a first-level analysis using the general linear model to examine the BOLD response to five event types: NoGo correct, RareGo correct, NoGo incorrect, RareGo incorrect, and Go incorrect. The NoGo correct event was indicative of correctly inhibiting a prepotent response while controlling for lapses in attention. Events were modeled as a delta regressor (0 s) and convolved with the canonical hemodynamic response function. Six intra-run motion parameters (*x, y, z*, roll, pitch, and yaw) were removed and included as first-level covariates, and a high-pass filter (128 s) was applied. The peak rIFG IC BOLD cluster for each participant was determined by examining the NoGo correct—RareGo correct BOLD contrast, which represents successful IC while controlling for lapses in attention and novelty detection.

#### Resting-state functional connectivity

During baseline and 20 min after receiving TBS, participants underwent an eyes-closed resting-state fMRI scan (duration = 10 min, volumes = 300). Images were preprocessed, denoised, and modeled with the CONN toolbox (version 21b, www.nitrc.org/projects/conn, RRID: SCR_009550). Preprocessing consisted of functional realignment and unwarping using b-spline interpolation (first EPI volume as reference image), slice-timing correction, outlier detection (framewise displacement > 0.9 mm or global BOLD signal > 5 standard deviations), and direct segmentation and normalization to MNI 152 space (anatomical resampled to 1 mm^3^ voxels; functional resampled to 1.5 mm^3^ voxels) using b-spline interpolation. Unsmoothed images were then put through a denoising anatomical component correction (aCompCor) pipeline to regress out BOLD signal confounds which included five cerebrospinal fluid and five white matter components, six motion parameters, scrubbing, task effects, despiking, and bandpass filtering (0.008, 0.09 Hz). Finally, voxel-wise, Fisher-transformed bivariate correlation coefficient (rZ) maps were calculated, and regions of interest (ROIs) were parcellated according to the Harvard-Oxford atlas (http://fsl.fmrib.ox.ac.uk/fsl/fslwiki/Atlases, RRID: SCR_001476). rsFC was assessed using the CONN ROI-to-ROI explorer.

### Theta-burst stimulation protocol

#### Neuronavigation

The Rogue Research Inc. Brainsight system was used to perform neuronavigation (67). Within each participant's native space, their anatomical image was co-registered to their peak rIFG BOLD cluster identified from the baseline IC GGNG task. Skin and full-brain curvilinear reconstructions were generated and anatomical landmarks (nasion, the tip of the nose, and left and right tragi) were created to enable registration between these images and each participant's head. The rIFG BOLD cluster was set as the spatial target, and the target coil trajectory was set. The same setup parameters were used for each TBS session (16). Neuronavigation target errors were recorded (Supplementary Table 3).

#### Stimulation equipment and parameters

The MagVenture MagPro X100 TMS Therapy System with a figure-8 Cool-B65 A/P coil at 80% RMT was used to administer TBS. Parameter estimation by sequential testing (PEST) was used to determine RMT at each TBS session by stimulating the motor cortex (68). The duration of the cTBS protocol was 40 s [three pulse bursts at 50 Hz repeated every 200 ms (5 Hz) and 600 total pulses], while iTBS was 190 s [3 pulse bursts at 50 Hz repeated every 200 ms (5 Hz) per train, 2 s per train, 20 trains, 10 s intertrain intervals, and 600 total pulses]. During TBS, participants were reclined in a comfortable chair and wore a mouthpiece and earplugs (16). RMT and treatment dosages were recorded (Supplementary Table 4).

### Blinding

To achieve researcher blinding, and to standardize TBS session duration and administration, this study had a dedicated TBS technician who was not involved with data collection. Excluding the TBS technician, researchers and participants were both blinded to treatment conditions.

### Outcome measures

All questionnaire data (cravings, side effects, and smoking) were collected electronically via REDCap. Questionnaires administered at the start and end of each session (cravings and side effects) were completed on a desktop computer in the laboratory, while questionnaires administered 24 h after each session (cravings, side effects, and smoking) were collected remotely via smartphone. For the remote assessments, participants were sent a text message containing a link that directed them to the questionnaires. Resting-state fMRI data were collected at the baseline session and 20 min after each TBS treatment. At the end of the final session, researchers that collected data and participants completed a study blind assessment.

#### Cravings

Differences in cravings were assessed by examining responses to both factors on the Questionnaire of Smoking Urges Brief (QSUB) (69, 70). QSUB factor one measures how strongly a participant desires and intends to smoke (henceforth, appetitive craving), while QSUB factor two measures how strongly a participant anticipates that smoking will provide relief from negative affect and urge to smoke (henceforth, withdrawal craving).

#### Smoking

Differences in smoking consumption were assessed by asking participants to report the number of cigarettes per day (CPD) they had consumed during the 24-h period after each session.

#### Resting-state functional connectivity

Differences in rsFC were assessed by having participants receive a resting-state fMRI scan at each session. A data-driven approach using the Harvard-Oxford atlas was used to parcellate the ROIs used for the rsFC analyses. ROI-to-ROI analyses were restricted to the right hemisphere, and only connections from the rIFG pars opercularis to the striatum and limbic system were examined. *A priori* striatal ROIs consisted of the subcallosal cingulate and right nucleus accumbens, while limbic ROIs consisted of the right posterior parahippocampal gyrus and right hippocampus. The rIFG pars opercularis was chosen as the primary cortical ROI because this region is associated with IC, regulation of craving, and was directly stimulated across the majority of participants in this study. The subcortical ROIs were selected based on previous literature indicating their involvement in rewarding smoking behaviors (33, 34).

#### Side effects

Differences in the total side effect symptoms were assessed by examining responses to the review of symptoms (ROS) questionnaire (71).

#### Blinding

The double-blind procedure was assessed by having researchers and participants complete a form indicating which order of treatment they believed was administered as well as provide a confidence rating on a scale from 1 to 10.

### Statistical analyses

To account for missing data and control for session start values, mixed modeling analysis of covariance (ANCOVA) was used to examine fixed effects of session on cravings and side effect symptoms at session end and post-24 h. Since the side effects outcome consisted of count data, a Poisson ANCOVA was used. An analysis of variance (ANOVA) was used to examine the fixed effects of session on smoking and rsFC. Linear regression was used to examine associations among smoking behaviors and if treatment-related change scores from baseline in rsFC (Δ: i/cTBS - baseline) were associated with smoking-related outcomes. Study blinding was assessed using a chi-square test. In all analyses, statistical significance was defined as *p* < 0.05 (two-sided). Graphical techniques such as boxplots, spaghetti plots, and scatterplots were used for the visualization of study outcomes.

## Results

### Cravings

#### Appetitive craving

**Figure 2:**
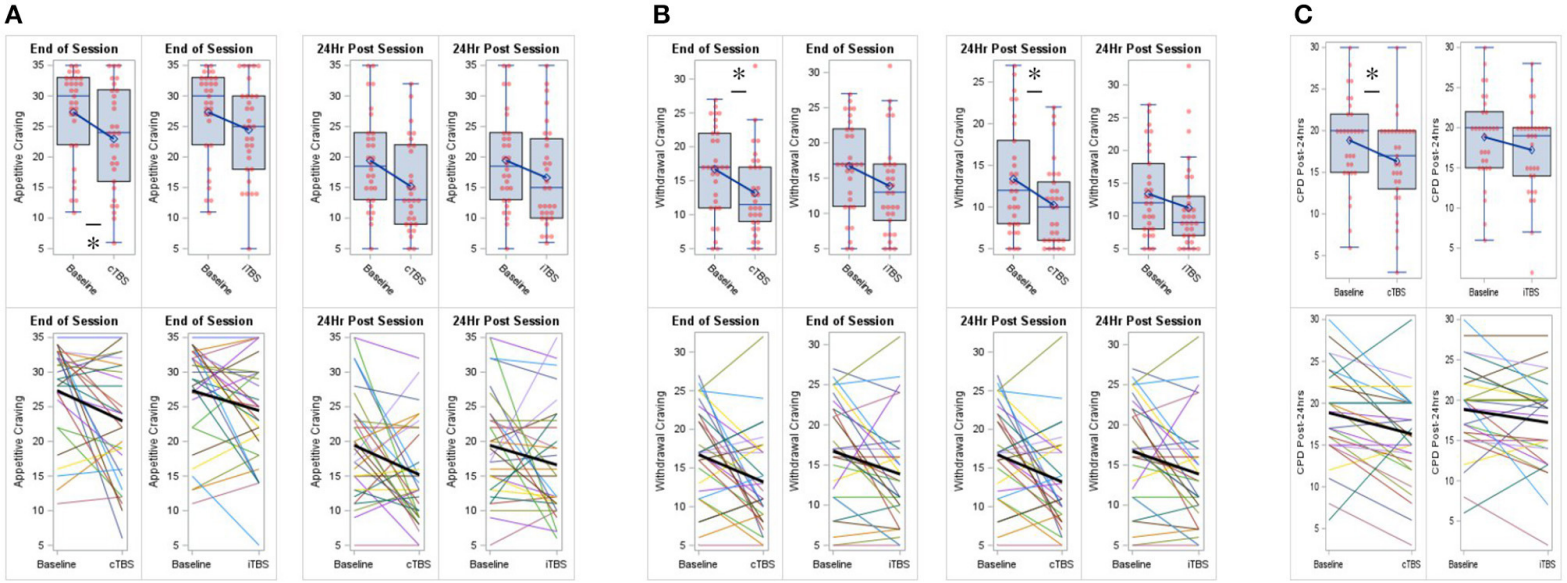
Continuous theta-burst stimulation (cTBS) to the right inferior frontal gyrus (rIFG) reduced cigarette cravings and smoking. **(A)** Compared to baseline, cTBS reduced appetitive craving at session end. **(B)** Compared to baseline, cTBS reduced withdrawal craving at session end and at post 24-h. **(C)** Compared to baseline, cTBS reduced smoking at post 24-h. iTBS did not significantly effect cigarette cravings or smoking. Data are presented with boxplots (top row) and spaghetti plots (bottom row). *p* < 0.05 (two-sided). *p < 0.05.

As compared to baseline (*M* = 27.35, *SD* = 7.33) when controlling for session start values, appetitive craving at session end was significantly reduced for cTBS (*M* = 22.97, *SD* = 8.48; *adj mean diff* = −4.09, *SE* = 2.00, *95% CI* = −8.02 to −0.17, *p* = 0.044, *Cohen's d* = −0.373), but not for iTBS (*M* = 24.45, *SD* = 7.79; *adj mean diff* = −2.95, *SE* = 1.98, *95% CI* = −6.82 to 0.93, *p* = 0.140, *Cohen's d* = −0.268) (*F*_*2*_ = 2.25, *p* = 0.118). As compared to baseline (*M* = 19.43, *SD* = 7.84) when controlling for session start values, appetitive craving at post-24 h was not significantly reduced for cTBS (*M* = 15.20, *SD* = 7.34; *adj mean diff* = −3.75, *SE* = 1.98, *95% CI* = −7.62 to 0.13, *p* = 0.061, *Cohen's d* = −0.346) or iTBS (*M* = 16.61, *SD* = 8.15; *adj mean diff* = −2.80, *SE* = 1.95, *95% CI* = −6.62 to 1.02, *p* = 0.154, *Cohen's d* = −0.258) (*F*_*2*_ = 1.95, *p* = 0.150) (Figure 2A).

#### Withdrawal craving

As compared to baseline (*M* = 16.71, *SD* = 6.49) when controlling for session start values, withdrawal craving at session end was significantly reduced for cTBS (*M* = 13.20, *SD* = 6.24; *adj mean diff* = −3.54, *SE* = 1.57, *95% CI* = −6.62 to −0.46, *p* = 0.027, *Cohen's d* = −0.412), but not for iTBS (*M* = 13.87, *SD* = 6.72; *adj mean diff* = −2.86, *SE* = 1.56, *95% CI* = −5.91 to 0.20, *p* = 0.070, *Cohen's d* = −0.329) (*F*_*2*_ = 2.88, *p* =0.062). As compared to baseline (*M* = 13.37, *SD* = 6.59) when controlling for session start values, withdrawal craving at post-24 h was significantly reduced for cTBS (*M* = 10.23, *SD* = 4.91; *adj mean diff* = −2.96, *SE* = 1.42, *95% CI* = −5.75 to −0.18, *p* = 0.040, *Cohen's d* = −0.380), but not for iTBS (*M* = 11.23, *SD* = 6.64; *adj mean diff* = −2.08, *SE* = 1.41, *95% CI* = −4.84 to 0.67, *p* = 0.142, *Cohen's d* = −0.265) (*F*_*2*_ = 2.31, *p* = 0.106) (Figure 2B).

### Smoking

As compared to baseline (*M* = 18.84, *SD* = 5.44), CPD at post-24 h were significantly reduced for cTBS (*M* = 16.32, *SD* = 5.53; *mean diff* = −2.52, *SE* = 0.84, *95% CI* = −4.236 to −0.796, *p* = 0.006, *Cohen's d* = −0.539), but not for iTBS (*M* = 17.23, *SD* = 5.55; *mean diff* = −1.61, *SE* = 0.82, *95% CI* = −3.277 to 0.051, *p* = 0.057, *Cohen's d* = −0.353) (*F*_*2, 29*_ = 4.972, *p* = 0.014) (Figure 2C).

### Resting-state functional connectivity

#### Fronto-striatal circuitry

**Figure 3:**
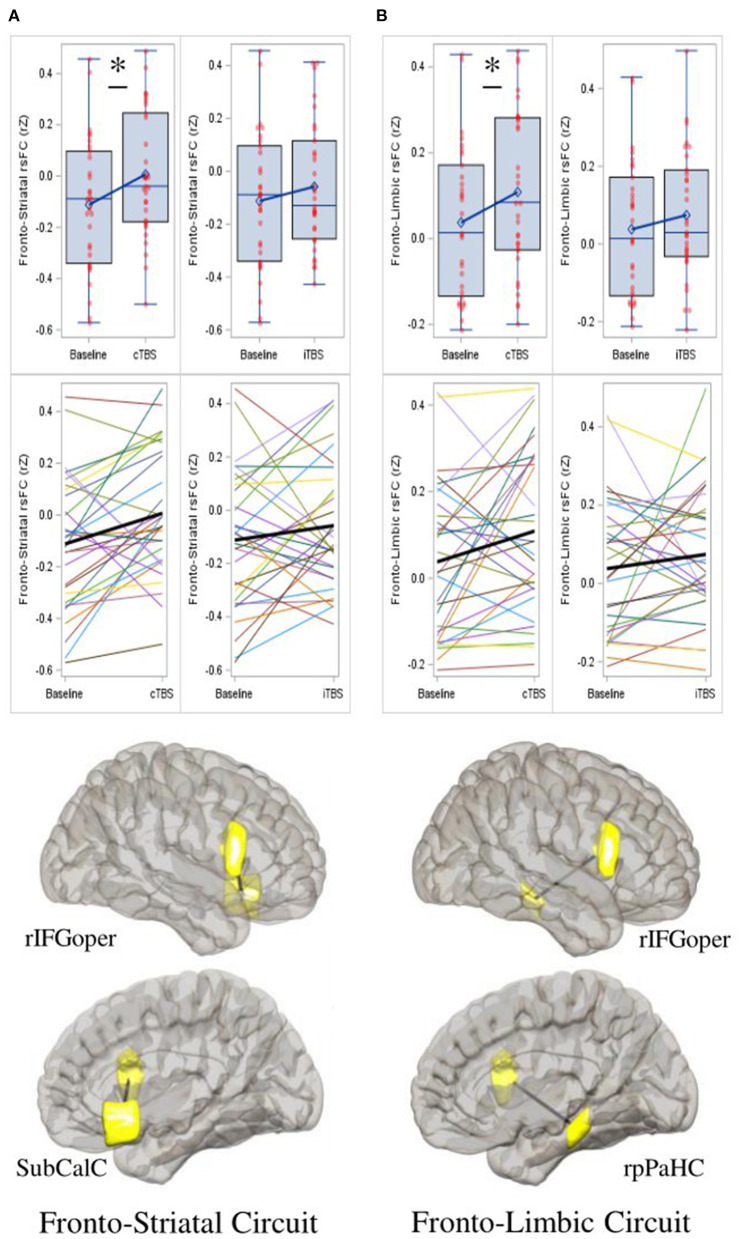
Continuous theta-burst stimulation (cTBS) strengthens fronto-striatal and fronto-limbic resting-state functional connectivity (rsFC). **(A)** Compared to baseline, cTBS strengthened rsFC between the right inferior frontal gyrus pars opercularis (rIFGoper) and subcallosal cingulate (SubCalC) (fronto-striatal circuit); and between **(B)** the rIFGoper and right posterior parahippocampal gyrus (rpPaHC) (fronto-limbic circuit). iTBS has no significant effects on either circuit. Data represent Fisher-transformed bivariate correlation coefficient (rZ) values between regions of interest (ROIs) defined by the Harvard-Oxford atlas. Depictions of ROIs are in yellow. Date are presented with boxplots (top row) and spaghetti plots (middle row). *p* < 0.05 (two-sided). *p < 0.05.

As compared to baseline (*M* = −0.112, *SD* = 0.26), rsFC between the rIFG pars opercularis and subcallosal cingulate was significantly increased for cTBS (*M* = 0.006, *SD* = 0.24; *mean diff* = 0.118, *SE* = 0.04, *95% CI* = 0.029 to 0.208, *p* = 0.011, *Cohen's d* = 0.530), but not for iTBS (*M* = −0.058, *SD* = 0.24; *mean diff* = 0.054, *SE* = 0.05, *95% CI* = −0.043 to 0.151, *p* = 0.263, *Cohen's d* = 0.194) (*F*_*2, 29*_ = 3.530, *p* = 0.042) (Figure 3A). No significant treatment effects were found on connectivity between the rIFG pars opercularis and right nucleus accumbens (Supplementary Table 5).

#### Fronto-limbic circuitry

As compared to baseline (*M* = 0.038, *SD* = 0.18), rsFC between the rIFG par opercularis and the right posterior parahippocampal gyrus was significantly increased for cTBS (*M* = 0.109, *SD* = 0.19; *mean diff* = 0.071, *SE* = 0.03, *95% CI* = 0.003 to 0.139, *p* = 0.042, *Cohen's d* = 0.425), but not for iTBS (*M* = 0.074, *SD* = 0.17; *mean diff* = 0.036, *SE* = 0.04, *95% CI* = −0.037 to 0.109, *p* = 0.317, *Cohen's d* = 0.162) (*F*_*2, 29*_ = 2.199, *p* = 0.129) (Figure 3B). No significant treatment effects were found on connectivity between the rIFG pars opercularis and the right hippocampus (Supplementary Table 5).

### Behavioral associations

Appetitive craving at session end and CPD at post-24 h were significantly positively associated for iTBS ($R_{adj}^{\text{2}}$ = 0.104, *F*_*1, 29*_ = 4.494, β = 0.261, *95% CI* = 0.009 to 0.513, *t* = 2.120, *p* = 0.043), while this association was not significant for cTBS ($R_{adj}^{\text{2}}$ = −0.009, *F*_*1, 28*_ = 0.729, β = 0.102, *95% CI* = −0.143 to 0.348, *t* = 0.854, *p* = 0.400) (Supplementary Figure 1A). Withdrawal craving at post-24 h and CPD at post-24 h were significantly positively associated for cTBS ($R_{adj}^{\text{2}}$ = 0.111, *F*_*1, 28*_ = 4.604, β = 0.427, *95% CI* = 0.019 to 0.835, *t* = 2.146, *p* = 0.041), while this association was not significant for iTBS ($R_{adj}^{\text{2}}$ = −0.010, *F*_*1, 29*_ = 0.716, β = 0.130, *95% CI* = −0.184 to 0.443, *t* = 0.846, *p* = 0.405) (Supplementary Figure 1B).

### Brain-behavior associations

#### Fronto-striatal circuitry and appetitive craving

cTBS-induced strengthening of fronto-striatal rsFC was marginally associated with decreased appetitive craving at session end ($R_{adj}^{\text{2}}=0.098$, *F*_*1, 28*_ = 4.160, β = −12.264, *95% CI* = −24.581 to 0.052, *t* = −2.040, *p* = 0.051), while this association was not present for iTBS ($R_{adj}^{\text{2}}$ = −0.025, *F*_*1, 29*_ = 0.277, β = −2.875, *95% CI* = −14.041 to 8.291, *t* = −0.527, *p* = 0.602) (Supplementary Figure 2A). cTBS-induced strengthening of fronto-striatal rsFC was significantly associated with decreased appetitive craving at post-24 h ($R_{adj}^{\text{2}}$ = 0.110, *F*_*1, 28*_ = 4.596, β = −11.087, *95% CI* = −21.681 to −0.493, *t* = −2.144, *p* = 0.041), while this association was not significant for iTBS ($R_{adj}^{\text{2}}$ = −0.022, *F*_*1, 29*_ = 0.343, β = −3.343, *95% CI* = −15.011 to 8.325, *t* = −0.586, *p* = 0.562) (Supplementary Figure 2B). No significant associations were found between fronto-striatal rsFC following cTBS or iTBS and withdrawal craving or CPD.

#### Fronto-limbic circuitry, withdrawal craving, and smoking

cTBS-induced strengthening of fronto-limbic rsFC was significantly associated with decreased withdrawal craving at post-24 h ($R_{adj}^{\text{2}}$ = 0.193, *F*_*1, 28*_ = 7.954, β = −12.509, *95% CI* = −21.594 to −3.424, *t* = −2.820, *p* = 0.009), while this association was not significant for iTBS ($R_{adj}^{\text{2}}$ = −0.028, *F*_*1, 29*_ = 0.187, β = 2.670, *95% CI* = −9.966 to 15.307, *t* = 0.432, *p* = 0.669) (Supplementary Figure 2C). cTBS-induced strengthening of fronto-limbic rsFC was significantly associated with reduced CPD at post-24 h ($R_{adj}^{\text{2}}$ = 0.105, *F*_*1, 29*_ = 4.520, β = −10.895, *95% CI* = −21.377 to −0.414, *t* = −2.126, *p* = 0.042), while this association was not significant for iTBS ($R_{adj}^{\text{2}}$ = 0.015, *F*_*1, 29*_ = 1.451, β = −6.091, *95% CI* = −16.433 to 4.251, *t* = −1.205, *p* = 0.238) (Supplementary Figure 2D). No significant associations were found between fronto-limbic rsFC following cTBS or iTBS and withdrawal craving at session end, or appetitive craving at any time point.

### Side effects

As compared to baseline and controlling for side effect symptoms reported at the start of each session, neither cTBS nor iTBS resulted in elevated reports of total symptoms at visit end. *Post-hoc* tests within each TBS condition comparing symptoms reported at session start to those reported at post-24 h revealed that neither cTBS nor iTBS had elevated symptoms (Supplementary Table 6; Supplementary Figure 3). No serious adverse events were reported.

### Blinding

The double-blind procedure was successful. Across sessions, neither the researchers that collected data [$\chi_{\left(2\right)}^{\text{2}}$ = 1.133, *p* = 0.567] nor the participants [$\chi_{\left(2\right)}^{\text{2}}$ = 0.619, *p* = 0.734] could correctly identify the order of TBS conditions administered.

## Discussion

This study assessed the acute effects of cTBS and iTBS to the rIFG on smoking behaviors and fronto-striatal-limbic rsFC within a community sample of nicotine-dependent adult cigarette smokers. The results demonstrated that cTBS reduced cigarette cravings and smoking and strengthened fronto-striatal-limbic rsFC. Furthermore, the magnitude of cTBS-induced change in fronto-striatal-limbic rsFC was associated with the attenuation of smoking behaviors. These findings provide initial support for applying cTBS to the rIFG to strengthen functional connectivity between cognitive control and reward circuitry, thereby attenuating craving and reducing smoking.

### Smoking behaviors

Although the construct of craving reflects a constellation of symptoms (72, 73), it represents a primary predictor of relapse (11). In this study, appetitive and withdrawal cravings were assessed (69), which may represent distinct mechanisms of craving, such as those based on positive and negative reinforcement, respectively (74). cTBS significantly reduced appetitive and withdrawal cravings immediately after treatment and these effects for withdrawal cravings persisted over a 24-h period. Moreover, cTBS produced a significant reduction in smoking over the 24-h period following treatment, and reduction in withdrawal craving was significantly positively associated with smoking fewer cigarettes over the 24-h period.

### Fronto-striatal rsFC and appetitive craving

cTBS strengthened rsFC between the rIFG pars opercularis and subcallosal cingulate (i.e., fronto-striatal circuit), and the magnitude of change in fronto-striatal rsFC was associated with reduced appetitive craving at 24 h post-treatment. These effects may be the result of rIFG cTBS remediating dysregulated top-down IC over appetitive craving elicited by the positively reinforcing effects of daily cigarette cue exposure (75). In studies among individuals that smoke cigarettes, BOLD activation in the subcallosal cingulate is associated with higher appetitive craving (76), whereas proactive downregulation of cigarette craving is associated with lower BOLD response in the subcallosal cingulate and higher BOLD response in the IFG (34), suggesting that these regions work together to regulate craving. Furthermore, an adult smoker's level of nicotine dependence has been shown to negatively correlate with IFG BOLD response during craving downregulation, suggesting that greater nicotine dependence is related to deficits in the capacity to regulate appetitive craving (36). Prior literature demonstrates that individuals with a SUD exhibit weaker fronto-striatal rsFC in comparison to controls (39, 42–46, 48, 77), and that fronto-striatal rsFC is negatively associated with addiction severity (43) and relapse vulnerability (39). Evidence also suggests that excessive glutamate in fronto-striatal circuitry contributes to maladaptive drug-seeking behavior (78). In theory, cTBS to the rIFG may modulate glutamatergic mediated fronto-striatal pathophysiology, improve glutamatergic tone, and help to restore regulatory control over motivationally relevant, yet maladaptive, cigarette cues. However, further research is needed to test this hypothesis.

### Fronto-limbic rsFC, withdrawal craving, and smoking

cTBS strengthened rsFC between the rIFG pars opercularis and posterior parahippocampal gyrus (i.e., fronto-limbic circuit), and the magnitude of change in fronto-limbic rsFC was associated with reductions in both withdrawal craving and smoking at 24 h post-treatment. In a similar line of reasoning as above, these effects may be the results of rIFG cTBS remediating top-down IC over withdrawal craving elicited by the negatively reinforcing emotional significance attributed to memories of past smoking episodes. It is well-known that the posterior parahippocampal gyrus is important for episodic memory (79), which includes memories of past drug use (80). Additionally, among smokers, smoking cues have been found to elicit increased BOLD response in both the parahippocampal gyrus and IFG (81). Prior literature demonstrates that among individuals with a SUD, fronto-limbic circuitry is weaker in comparison to non-SUD controls (39, 43, 45, 47, 49, 50, 77), and that weaker fronto-limbic rsFC is associated with addiction severity (50) and relapse vulnerability (39). Evidence also demonstrates that chronic drug use modifies circuitry underlying learning and memory (82). In theory, cTBS to the rIFG may treat dysregulated fronto-limbic circuitry function and improve regulatory control over the motivational significance attributed to recalling past smoking episodes. Future research that examines the effects of TBS on associative learning processes may shed light on how neuromodulation of fronto-limbic circuitry mediates learning, memory, and smoking.

### Brain-behavior associations

The observed associations between brain and behavioral outcomes following cTBS provide further support for the distinction of separate craving mechanisms in nicotine-dependent adults. To summarize, cTBS-induced strengthening of fronto-striatal rsFC was significantly associated with reduced appetitive craving, while strengthening of fronto-limbic rsFC was significantly associated with reduced withdrawal craving and smoking. These findings are consistent with the extant literature, suggesting that positive reinforcement components of craving are largely mediated by dysregulated fronto-striatal circuitry; whereas negative reinforcement components of craving are largely mediated by fronto-limbic circuitry (74, 75, 83). Moreover, the results from the current study demonstrating that rIFG cTBS modulates both circuits in a corresponding craving process-specific manner are intriguing and provide initial support for conducting a larger-scale sham-controlled study.

### Limitations

Despite numerous strengths of this study including a translational clinical neuroscience model-based approach, well-controlled design, and successful blind and remote assessments, there are notable limitations. First, the current study compared two active TBS treatments to a baseline session without a sham condition. Second, only one treatment session was administered per TBS protocol, thus limiting the ability to evaluate the durability of the observed outcomes. Third, the remote assessments relied strictly on self-report and did not include biochemical verification; however, participants were encouraged to report honestly and were informed they would be compensated for reporting, not for values reported. Fourth, the TBS treatments were delivered at 80% RMT, which is a relatively low dose. Future research following up on this report should consider addressing these limitations in order to further determine the mechanisms and potential value of rIFG cTBS for treating addiction pathophysiology. Additionally, future studies examining dose-response parameters may be warranted.

## Conclusion

Current study findings demonstrating that a single dose of rIFG cTBS at 80% RMT strengthens fronto-striatal-limbic rsFC and is associated with reductions in cravings, and smoking elucidates a neural circuit model that may be further examined for improving smoking cessation outcomes in adults with nicotine dependence. These findings are intriguing because the rIFG is a novel understudied cortical target in addiction therapy, an accessible cortical target for neuromodulation, and may have effects on dissociable neural pathways subserving response inhibition and incentive salience, which are two core neurocognitive deficits underlying addiction. Despite prior theoretical models of the dissociable effects of cTBS and iTBS, the current study results bolster the rationale for further examination of the effects of repeated rIFG cTBS for treating addiction pathophysiology and promoting smoking cessation.

## Data availability statement

The raw data supporting the conclusions of this article will be made available by the authors, without undue reservation.

## Ethics statement

The studies involving human participants were reviewed and approved by Institutional Review Board of the University of Missouri - Columbia. The patients/participants provided their written informed consent to participate in this study.

## Author contributions

SU: conceptualization, data curation, formal analysis, investigation, methodology, visualization, writing—original draft, and writing—reviewing and editing. AB: data curation, visualization, and writing—reviewing and editing. MG: formal analysis and visualization. EG: writing—reviewing and editing. BF: conceptualization, formal analysis, investigation, methodology, project administration, resources, supervision, validation, visualization, writing—original draft, and responsible for securing funding. All authors contributed to the article and approved the submitted version.
