# Supplementary material for: Right inferior frontal gyrus theta-burst stimulation reduces smoking behaviors and strengthens fronto-striatal-limbic resting-state functional connectivity: a randomized crossover trial
Source: Front Psychiatry. 2023 Jun 28;14:1166912. doi: 10.3389/fpsyt.2023.1166912 (PMC10338839; doi:10.3389/fpsyt.2023.1166912)
Supplement: Supplementary file 1 [file Data_Sheet_1.pdf]

Supplementary Data for

Right inferior frontal gyrus theta-burst stimulation reduces smoking behaviors and strengthens fronto-striatal-limbic resting-state functional connectivity: a randomized crossover trial

Spencer Upton *et al.*

* Corresponding author. Email: froeligerb@health.missouri.edu

This file includes:

Tables S1 to S6
Figures S1 to S3

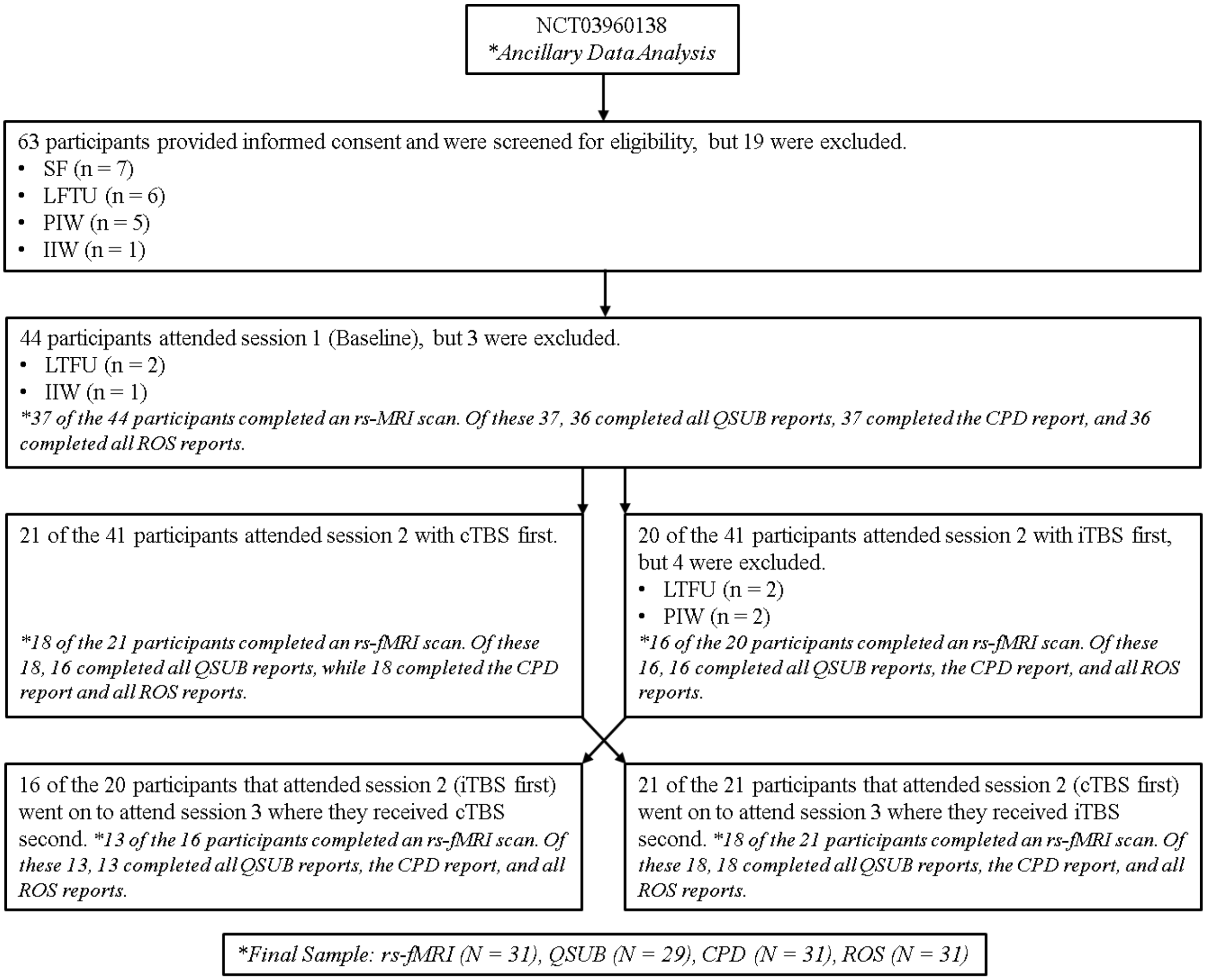

Table S1. CONSORT Flow Diagram. Participants in this study were part of a larger clinical trial that examined the effects of intermittent and continuous theta-burst stimulation (i/cTBS) to the right inferior frontal gyrus (rIFG) on brain and behavior outcomes in individuals with nicotine dependence (ClinicalTrials.gov Identifier: NCT03960138). Ancillary aims and analyses presented here were focused on examining TBS effects on resting-state functional connectivity and smoking-related behaviors. Abbreviations: screen fails, SF; lost to follow up, LTFU; participant-initiated withdrawal, PIW; investigator-initiated withdrawal, IIW; resting-state functional magnetic resonance imaging, rs-fMRI; questionnaire of smoking urges brief, QSUB; cigarettes per day, CPD; review of symptoms, ROS.

Session start	CO Levels (M, SD)	QSUB Appetitive Craving (M, SD)	QSUB Withdrawal Craving (M, SD)
Baseline	26.39, 9.48	11.74, 5.05	8.71, 3.51
iTBS	25.61, 12.20	11.90, 6.00	8.74, 4.92
cTBS	25.00, 13.06	10.52, 5.14	8.65, 4.31
1-Way Repeated Measures ANOVA	$F_{2,29}$ = 0.218, *p* = .806	$F_{2,29}$ = 0.994, *p* = .382	$F_{2,29}$ = 0.020, *p* = .980
Pairwise comparisons *p<.05 (two-sided)	Base vs. cTBS: *p* = .528 Base vs. iTBS: *p* = .708	Base vs. cTBS: *p* = .245 Base vs. iTBS: *p* = .879	Base vs. cTBS: *p* = .938 Base vs. iTBS: *p* = .968
Abbreviations: carbon monoxide, CO; questionnaire of smoking urges brief, QSUB.			

Table S2. Session Start Carbon Monoxide (CO) and Cigarette Cravings Results. CO and cigarette cravings were recorded at the start of each session. There were no significant differences in CO or cigarette cravings at the start of each session which confirmed that participants began each session with equivalent levels of nicotine satiety.

Session	Target Error, millimeters (M, SD)
iTBS	1.18, 0.85
cTBS	1.37, 1.23
Paired t-test *p<.05 (two-sided)	t_{30} = -0.816, *p* = .421

Table S3. Brainsight Neuronavigation Target Error Results. The Brainsight neuronavigation system recorded how far off in millimeters each theta-burst stimulation (TBS) treatment was from the target. The average target error for each TBS condition was less than 1.5 mm, and there were no significant differences between conditions.

Session	Resting Motor Threshold (RMT) (Amplitude: M, SD)	TBS Dosage at 80% RMT (Amplitude: M, SD)
iTBS	57.06, 9.72	45.71, 7.72
cTBS	57.68, 10.63	46.23, 8.53
Paired t-test *p<.05 (two-sided)	t_{30} = -0.827, *p* = .415	t_{30} = -0.837, *p* = .409

Table S4. Resting Motor Threshold (RMT) and Treatment Dosage Results. RMT was calculated at each TBS condition. TBS dosage was calculated as 80% RMT. There were no significant differences in RMT or treatment dosage across TBS conditions.

**Session**	**rIFG oper to rNAcc (M, SD)**	**rIFG oper to rHipp (M, SD)**
Baseline	-0.050, 0.143	0.015, 0.200
iTBS	-0.052, 0.142	0.005, 0.154
cTBS	-0.027, 0.233	0.040, 0.225
1-Way Repeated Measures ANOVA	$F_{2,29}$ = 0.216, p = .807	$F_{2,29}$ = 0.358, p = .702
Pairwise comparisons *p<.05 (two-sided)	Base vs. cTBS: p = .538<br>Base vs. iTBS: p = .958	Base vs. cTBS: p = .501<br>Base vs. iTBS: p = .776
Abbreviations: right inferior frontal gyrus pars opercularis, rIFGoper; right nucleus accumbens, rNAcc; right hippocampus, rHipp.		

Table S5. Extended Fronto-Striatal-Limbic Resting-State Functional Connectivity (rsFC) Results. Planned analyses also examined rsFC between the right inferior frontal gyrus pars opercularis (rIFG oper) and the right nucleus accumbens (rNAcc); and rsFC between the rIFGoper and right hippocampus (rHipp). Neither connection was significantly modulated by TBS.

**Session**	**ROS session start (M, SD)**	**ROS session end (M, SD)**	**ROS 24 hours post-session (M, SD)**	**Post-hoc paired t-test: Start vs. Post 24 hours**
Baseline	4.07, 3.79	3.29, 3.43	2.23, 2.47	Mean diff = -1.84, SD = 3.23, t = -3.174, p = .003
iTBS	2.61, 3.44	2.74, 3.79	2.48, 2.93	Mean diff = -0.13, SD = 2.51, t = -0.286, p = .777
cTBS	2.65, 3.26	2.84, 3.74	2.61, 3.13	Mean diff = -0.03, SD = 1.70, t = -0.105, p = .917
Poisson regression controlling for session start		χ^2 (2) = 1.360, p = .508	χ^2 (2) = 9.50, p = .009	
Wald chi-square p-value, *p<.05 (two-sided)		Base vs. cTBS: p = .257<br>Base vs. iTBS: p = .445	Base vs. cTBS: p = .005<br>Base vs. iTBS: p = .016	
Abbreviations: review of symptoms questionnaire, ROS.				

Table S6. Side Effect Results. Treatment-related side effects were evaluated by a review of symptoms (ROS) questionnaire at the start and end of each session, and at 24 hours after each session. Compared to baseline, neither cTBS nor iTBS resulted in elevated total symptoms at session end, but symptoms for both TBS conditions were elevated at post-24 hours [75]. Although symptoms were significantly elevated at post-24 hours, no serious adverse events were reported. However, post-hoc testing within each session revealed that neither cTBS nor iTBS had any difference in symptoms reported at session start compared to those reported at post-24 hours.

S1A

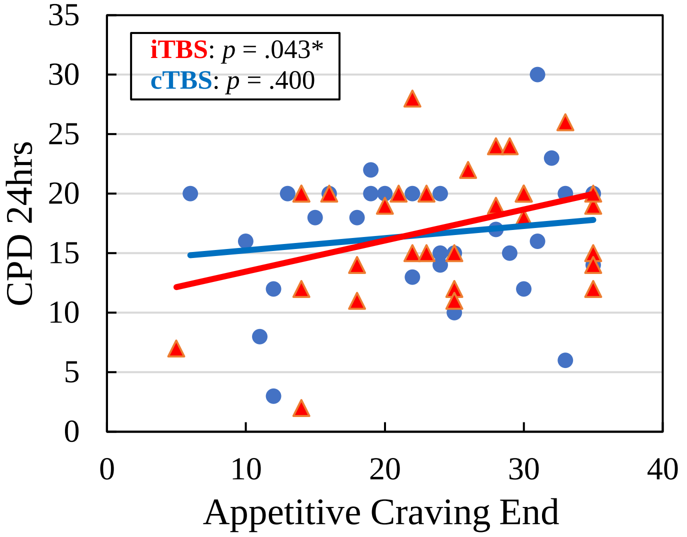

S1B

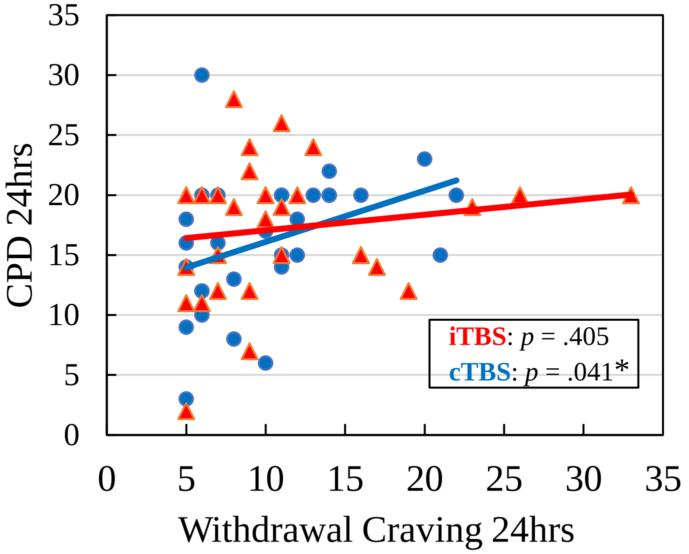

Figure S1. Behavioral associations following continuous theta-burst stimulation (cTBS) and intermittent TBS (iTBS). Linear regression analyses were conducted examining associations among smoking behaviors. (A) Appetitive craving at session end was positively associated with cigarettes per day (CPD) at 24 hours post-session for iTBS, but not for cTBS. (B) At 24 hours post-session, withdrawal craving, and CPD were positively associated for cTBS, but not for iTBS. $p < .05$ (two-sided). $*p < .05$.

S2A

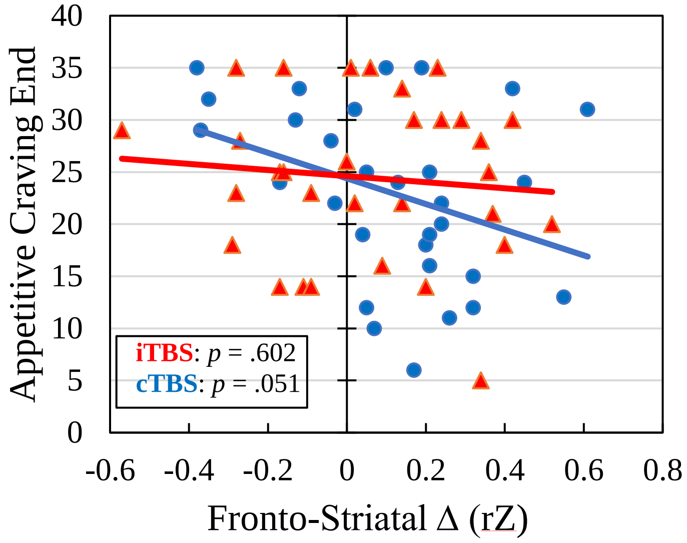
iTBS: p = .602
cTBS: p = .051
Appetitive Craving End
Fronto-Striatal Δ (rZ)

S2B

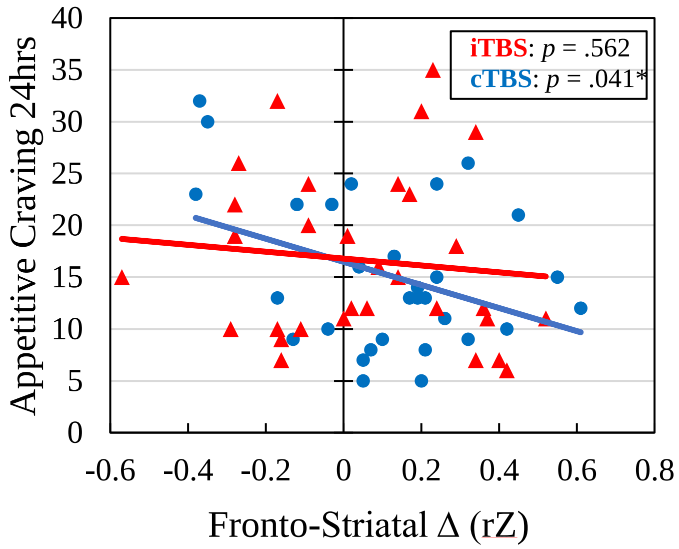
iTBS: p = .562
cTBS: p = .041*
Appetitive Craving 24hrs
Fronto-Striatal Δ (rZ)

S2C

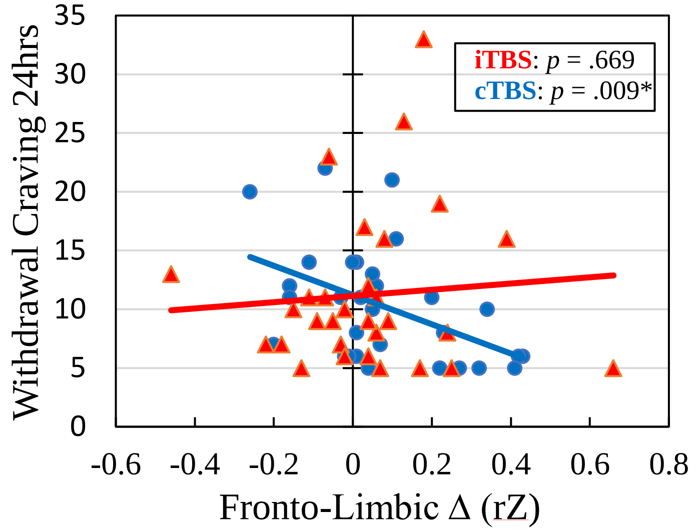

S2D

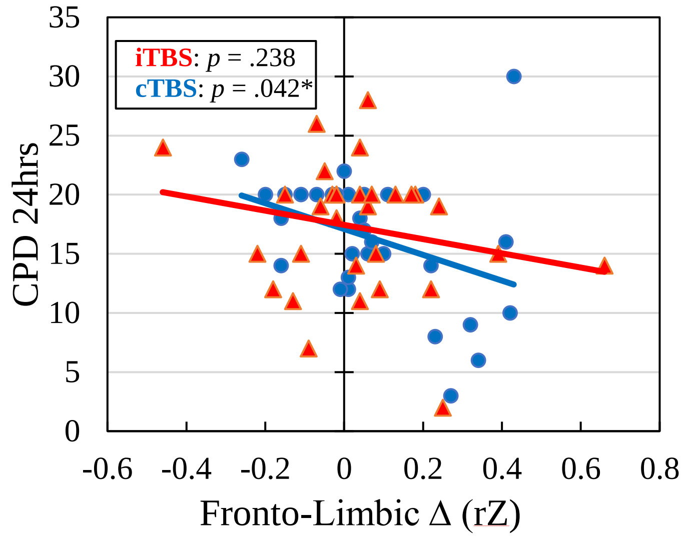

Figure S2. Strengthening of fronto-striatal-limbic resting-state functional connectivity (rsFC) following continuous theta-burst stimulation (cTBS) is associated with reductions in smoking behaviors. Linear regression analyses were conducted examining associations between fronto-striatal-limbic rsFC delta scores (Δ: c/iTBS - Baseline) and smoking behaviors. (A) Fronto-striatal rsFC Δ was marginally associated with appetitive craving at session end for cTBS but not iTBS. Moreover, cTBS resulted in the following significant associations: (B) fronto-striatal rsFC Δ and appetitive craving at post-24 hours; (C) fronto-limbic rsFC Δ and withdrawal craving at post-24 hours; and (D) fronto-limbic rsFC Δ and CPD at post-24 hours. These associations were not significant for iTBS. $p < .05$ (two-sided). *$p < .05$.

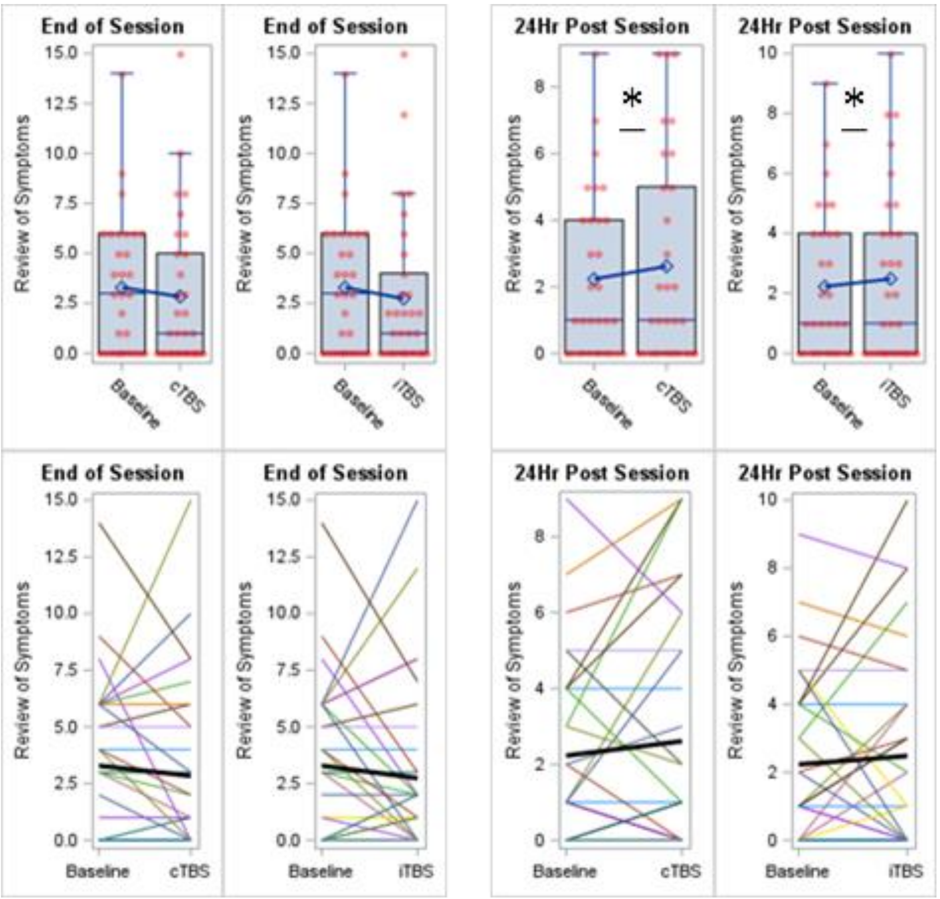

Figure S3. Boxplots and spaghetti plots for side effect results. As compared to baseline and controlling for side effect symptoms reported at the start of each session, neither cTBS nor iTBS resulted in elevated reports of total symptoms at session end. However, at post-24 hours, both TBS conditions had significantly elevated self-reported symptoms. Post-hoc tests within each TBS condition comparing symptoms reported at session start to those reported at post-24 h revealed that neither cTBS nor iTBS had elevated symptoms. No serious adverse events were reported. $p < .05$ (two-sided). $^*p < .05$.
